# Ovarian damage following surgery for endometriomas, 20 years later: did awareness improve the situation?

**DOI:** 10.1007/s00404-025-08039-x

**Published:** 2025-05-16

**Authors:** Dalila Invernici, Gianfranco Fornelli, Marco Reschini, Irene La Vecchia, Paola Vigano’, Edgardo Somigliana, Paolo Vercellini, Laura Benaglia

**Affiliations:** 1 Università degli Studi di Milano, Milan, Italy; 2 Department of Obstetrics and Gynecology, Fondazione IRCCS Ca’ Granda Ospedale Maggiore Policlinico, Milan, Italy; 3 Department of Clinical Sciences and Community Health, Academic Center for Research on Adenomyosis and Endometriosis, Università degli Studi di Milano, Milan, Italy

**Keywords:** Endometriosis, Endometrioma, Ovarian reserve, Ovarian failure, Surgery, IVF

## Abstract

**Background:**

Early reports from the beginning of this century highlighted significant ovarian damage following laparoscopic stripping of endometriomas. During the last 2 decades, the raised awareness of the possible detrimental effects of surgery has boosted the attention of surgeons on sparing ovarian reserve. Giving this increased consciousness on this issue, in this real-life study, we aimed to assess whether the surgically-related damage to the ovaries has been reduced over the years.

**Materials and methods:**

Ovarian reserve was assessed by comparing ovarian response during stimulation for IVF in women who previously underwent surgical treatment for unilateral endometriomas. This study design allows intra-patient comparisons, limiting confounders. In addition, this same design was used in the past and allows therefore to compare findings with those obtained 2 decades ago. The primary aim was the frequency of non-response among operated gonads.

**Results:**

One hundred seven female patients with unilateral endometrioma surgery who underwent IVF/ICSI cycles were eligible for the study. The mean ± SD diameter of the excised endometriomas was 46 ± 20 mm. Most women underwent cyst stripping while drainage and ablation was used in only three subjects. The median [IQR] number of follicles in the operated and contralateral gonads was 3 [1–5] and 7 [5–9], respectively (*p* < 0.001). Absence of follicular growth was observed in 19 operated ovaries (18%, 95% CI 12–26%). The magnitude of the damage remained unchanged, if not worse, when compared to what observed 2 decades ago.

**Conclusions:**

Ovarian reserve continues to be significantly compromised after surgery for endometriomas. Further research is urgently needed to better understand the underlying mechanisms and to refine surgical techniques aimed at minimizing this damage.

## Introduction

Initial reports in the early years of this century highlighted that ovaries are significantly harmed following laparoscopic stripping of endometriomas [1, 2]. Following several studies on this topic, systematic reviews and meta-analyses confirmed this evidence. Serum anti-mullerian hormone (AMH), the most reliable biomarker of ovarian reserve, was shown to be significantly reduced after surgery [3, 4]. In ART settings, responsiveness to stimulation was halved in operated ovaries [5] and in one out of eight cases the gonad was definitely compromised and did not respond at all [6]. The damage was inevitably worse after surgery for bilateral endometriomas [5] and, even if rare, cases of post-surgical ovarian failure could be identified [7].

During the last 2 decades, the raised awareness of the possible detrimental effects of surgery has boosted the attention of surgeons on the importance of ovarian reserve. Indications to surgery have progressively changed. Surgery is now advocated only for larger cysts (more than 4 cm in diameter), pain refractory to hormonal treatments and doubtful ultrasound findings that cannot reliably rule out malignancy [8]. In addition, a plethora of surgical arrangements and new type of interventions were proposed to limit the damage. They include a more careful technique with lower use of electric diathermy coagulation [9], drainage and laser vaporization of the inner cyst layer [10, 11], use of hemostatic sutures or sealants rather than diathermy coagulation to achieve hemostasis [12, 13], a combined technique of stripping and vaporization [14, 15], and the drainage of the cyst content with or without local injection of sclerotic agents [16, 17]. Results are however not univocal or not robust yet, and the gold standard remains the laparoscopic stripping [18, 19].

The real-life impact of all these efforts has not been studied. Most recent available meta-analyses continue to provide evidence on a relevant damage to ovarian reserve but included studies inevitably refer to women operated 10 or more years ago [20, 21] and inferences on the possible improvements in current real-life clinical practice cannot be extrapolated. To address this issue, we repeated the study performed 20 and 13 years ago in our clinic [2, 6]. In those studies, we selected women previously operated for unilateral endometriomas who underwent IVF and compared response to ovarian stimulation between the operated and the contralateral intact gonads. This study design allows to obtain reliable information on ovarian reserve since response to stimulation is considered a valuable surrogate measurement. In addition, it has the important advantage of abolishing inter-individual variability, strengthening statistical power (paired analyses can be drawn) and protect findings from most confounders.

## Materials and methods

Data from IVF-ICSI cycles performed at the Infertility Unit of the Fondazione IRCCS Ca’ Granda Ospedale Maggiore Policlinico between January 2014 and December 2022 were retrospectively reviewed. Inclusion criteria were as follows: (1) previous laparoscopic excision of one or more unilateral endometriotic ovarian cysts, (2) availability of a detailed description of the surgical intervention, (3) age ≤ 42 years at the time of ovarian stimulation, (4) > 3 developed follicles > 11 mm in the non-operated ovary (to exclude women with low ovarian reserve independently to surgery for endometriosis). The presence of a recurrent endometrioma at the time of IVF was not an exclusion criterion because we did not aim to exclude less compromised gonads [22]. Exclusion criteria were as follows: (1) previous surgery for non-endometriotic ovarian cysts, (2) presence of non-endometriotic ovarian cyst at the time of the procedure, (3) previous surgery for bilateral endometriomas, (4) ≥ 2 previous surgeries for ovarian endometriomas (even if in the same side), (5) surgery performed prior to 2010. Patients were selected regardless of the time between surgery and the IVF cycle. Information regarding the employed surgical technique was obtained from surgical, sonographic, and pathologic records. Only results observed in the first IVF cycle were considered. Approval for the study was obtained by the local Institutional Review Board. All patients referred to our Unit during the study period gave their informed consent to the use of their clinical data for research purposes.

Ovarian stimulation, oocyte retrieval and embryological management were standardized and performed as previously described [23]. Briefly, the protocol of stimulation, the initial dose of gonadotropins, as well as dose adjustments during treatment, were chosen on a case-by-case basis, according to patients’ characteristics. Dosage of gonadotropins was decided based on age, hormonal tests, ultrasound characteristics of the ovaries. In all cases, follicular growth was monitored by serial transvaginal ultrasonography. Ovulation was triggered with human chorionic gonadotropin (hCG) or GnRH agonists when two or more leading follicles had mean diameter ≥ 18 mm. On the day of trigger, a detailed transvaginal ultrasound scan was performed to record number and diameter of all follicles with a mean diameter ≥ 11 mm. Diameter of follicles was calculated as the mean of three perpendicular diameters. This information was routinely recorded separately for the two ovaries. Transvaginal oocyte retrieval was performed 36 h after hCG administration and transfer of fresh embryos 2–3 days later. In case of contraindications to fresh embryo transfer (such as conditions at risk of subsequent Ovarian Hyperstimulation Syndrome — OHSS, or elevated serum progesterone), embryos and/or oocytes were frozen and used subsequently. Supernumerary embryos could be frozen and transferred in subsequent cycles. In most cases, only one embryo is transferred at a time (and never more than two).

All female patients underwent in-depth sonographic evaluations prior to initiate the cycle. The diagnosis of recurrent endometrioma was performed according to established criteria and had to be documented on at least two occasions and at least two menstrual cycles apart [24]. Clinical pregnancy was defined as the ultrasonographic demonstration of an intrauterine gestational sac and a viable embryo 4–5 weeks after embryo transfer.

The primary outcome was the rate of operated ovaries that did not show any developed follicle. The secondary outcome considered was the number of follicles with a mean diameter ≥ 11 mm at the time of triggering. The sample size was calculated hypothesizing that this rate was unchanged compared to previous evidence, i.e., about 10% [6, 7]. We aimed to obtain a rate with a precision of ± 5%. On these bases, the number of women to be included was about 140 (https://www.openepi.com). We estimated that the period 2014–2022 could allow us to achieve this goal.

Analysis of the data was performed using the Statistics Package for Social Sciences (SPSS 26.0, Chicago, IL., USA). A binomial distribution model was used to calculate the 95% Confidence Interval (CI) of proportions. The number of follicles per ovaries was compared using non-parametric Wilcoxon test for paired data. The proportion of silent gonads was compared using a *χ*^2^ test with one degree of freedom. Probability values < 0.05 were considered statistically significant.

## Results

**Fig. 1:**
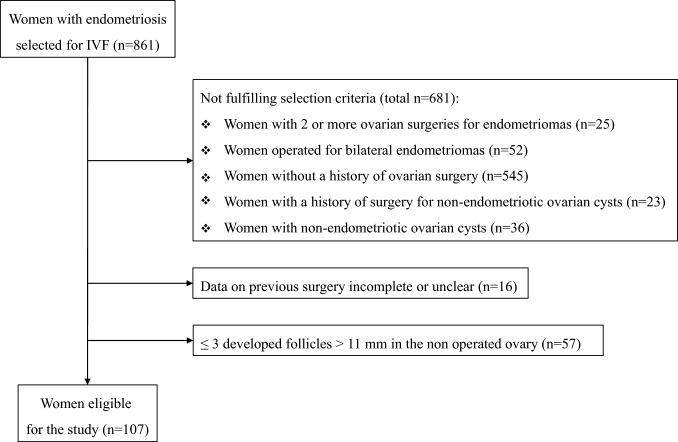
Flowchart of patients’ selection. In the end, 107 were eligible. Of relevance is the high proportion of women with endometriosis who underwent IVF without a history of surgery for endometriomas

During the study period, 861 women with endometriosis underwent IVF. The flow chart of the selection process is shown in Fig. 1. One hundred seven women were eligible for the study. Baseline clinical characteristics of the selected women are shown in Table 1. Recurrence of endometriomas at the time of IVF was observed in 24 (22%) women. Details on the past surgical intervention are presented in Table 2. The mean ± SD diameter of the excised endometriomas was 46 ± 20 mm. It exceeded 40 mm in 59% of cases. The vast majority of women underwent cyst stripping. Finally, cycle outcome is presented in Table 3. Cumulative live birth rate was 33%.

**Table 1:** Baseline clinical characteristics of the selected women (*n* = 107)

Characteristics	*N*. (%) or Mean ± SD or Median [IQR]
Age (years)	34.7 ± 3.6
BMI (kg/m^2^)	21.3 ± 2.8
Smoking	12 (11%)
Previous pregnancies	16 (15%)
Duration of infertility (years)	2.7 ± 1.5
Previous IVF cycles	2 (2%)
Previous gynecological surgery (excluding endometriosis)	26 (24%)
Male infertility associated	32 (30%)
Recurrence of endometriomas	24 (22%)
AMH (ng/mL)	1.8 ± 1.2
Day 3 serum FSH (IU/ml)	8.6 ± 3.5
Total AFC	8 ± 5

*BMI* Body Mass Index, *AMH* anti-mullerian hormone, *FSH* Follicle Stimulating Hormone, *AFC* Antral Follicle Count, *SD* Standard Deviation, *IQR* Interquartile Range

**Table 2:** Characteristics of the selected women at the time of surgery (*n* = 107)

Characteristics	*N*. (%) or Mean ± SD or Median [IQR]
Age (years)	31 ± 5
Time between surgery and IVF (years)	4.6 ± 3.6
Surgical unit	
Internal (our hospital)	43 (40%)
External	64 (60%)
Surgical approach	
Laparoscopy	92 (93%)
Laparotomy	8 (7%)
Type surgery	
Cyst stripping	104 (97%)
Cyst wall ablation	3 (3%)
Side	
Right	48 (45%)
Left	59 (55%)
Diameter (mm) ^a^	
Mean	47 ± 20
*N*. ≥ 4 cm	62 (59%)
Concomitant treatment of DIE	15 (14%)
ASRM Stage	
Stage III	69 (65%)
Stage IV	37 (35%)

*IQR* Interquartile Range, *DIE* Deep Infiltrative Endometriosis, *ASRM* American Society for Reproductive Medicine

^a^If more than one endometrioma was present, the data refers to the largest one

**Table 3:** IVF cycle outcome in the selected women (*n* = 107)

Characteristics	Mean ± SD or Number (%)
Protocol of stimulation	
GnRH antagonist	80 (75%)
Long protocol	16 (15%)
Flare up protocol	11 (10%)
Duration of stimulation (days)	9.1 ± 1.8
Total dose of gonadotropins (IU)	2161 ± 727
Total number of developped follicles (> 11 mm)	11.1 ± 5.6
Number of oocytes retrieved	7.1 ± 5.1
Number of oocytes suitable for insemination	5.2 ± 3.2
Total number of cleavage embryos obtained	3.7 ± 2.7
Cumulative clinical pregnancies	37 (35%)
Cumulative live births	35 (33%)

**Fig. 2:**
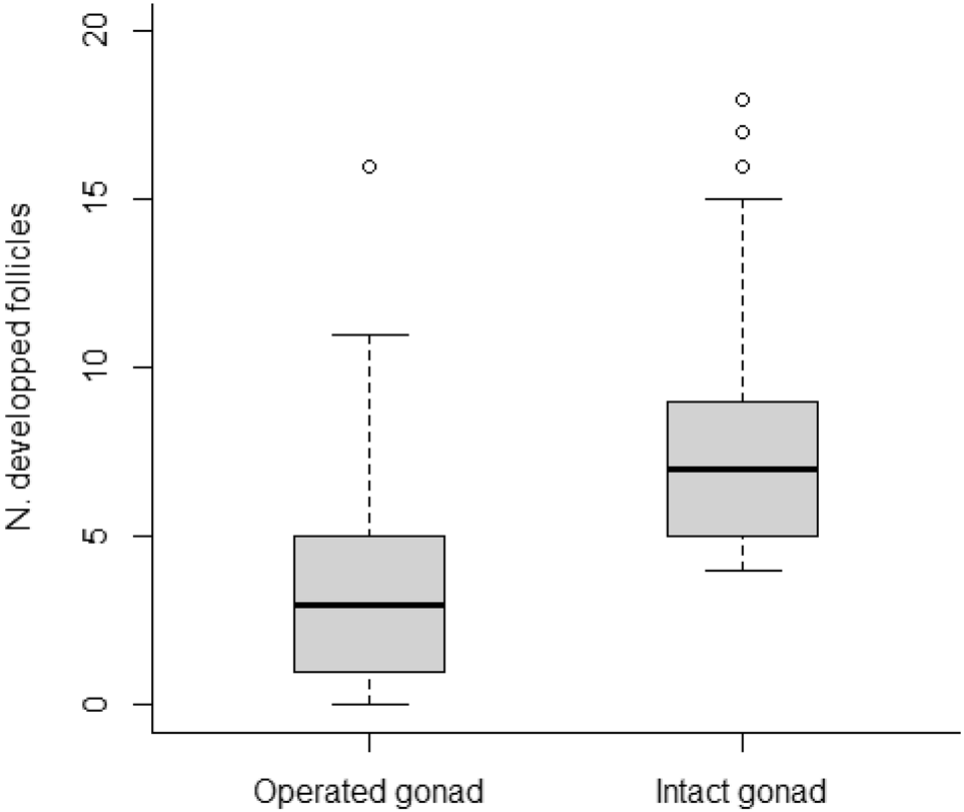
**N**umber of developed follicles (diameter > 10 mm) at the time of trigger in the operated and contralateral intact gonads

The median [IQR] number of follicles in the operated and contralateral gonads was 3 [1–5] and 7 [5–9], respectively (*p* < 0.001) (Fig. 2). Absence of follicular growth was observed in 19 operated ovaries. The frequency (95% CI) of nonresponsive ovaries following surgery was 18% (95% CI 12–26%). These analyses were repeated in subgroups of women according to the site where they were operated (locally or in other hospitals), the diameter of the excluded endometrioma (< or ≥ 4 cm) and whether they had a recurrence at the time of IVF. Results are presented in Table 4. We failed to identify a subgroup of markedly increased risk. Subgroup analyses according to the surgical technique used could not be done because only three women had cyst wall ablation.

**Table 4:** Subgroup analyses on the impact of endometrioma treatment on ovarian response

Subgroup	*N*	N. developped follicles			Compromised operated gonad	
		Operated gonad	Intact gonad	*p*	*N*	% (95% CI)
Surgical unit						
Internal (our hospital)	43	3 [1–5]	7 [5–10]	< 0.001	6	14% (7–27%)
External	64	2 [1–5]	7 [5–9]	< 0.001	13	20% (12–32%)
Diameter of the removed cyst						
< 4 cm	43	3 [1–5]	7 [5–10]	< 0.001	9	21% (11–35%)
≥ 4 cm	64	3 [1–5]	7 [5–9]	< 0.001	10	16% (9–26%)
Endometrioma recurrence						
Yes	24	4 [1–7]	7 [5–9]	< 0.001	3	13% (4–31%)
No	83	2 [1–5]	7 [5–9]	< 0.001	16	19% (12–29%)
All cohort	107	3 [1–5]	7 [5–9]	< 0.001	19	18% (12–26%)

A compromised gonas was defined as the absence of any follicular growth

## Discussion

Our findings suggest that the rate of severe ovarian damage following surgery for endometriomas has not improved over the years. Lack of follicular growth was indeed recorded in 18% of cases (95% CI 12–25%), thus not lower than what observed more than a decade ago in the same context (13%, 95% CI 7–21%) [6]. In addition, we confirmed a markedly lower response in operated gonads, again in line with previous evidence in our and other centres [25]. At first glance, our results are disappointing. Nonetheless, they merit some more in-depth comments.

First, as alluded above, the grown awareness on the possible detrimental effects of surgery on ovarian reserve had two major consequences. On one hand, there was a plea for a more cautious and meticulous surgical technique, limiting as much as possible the coagulation of the ovaries during and after the stripping. Some authors even advocated a change in the surgical technique to reduce the harm. A combined approach of excision and ablation was among the first surgical strategies proposed for the treatment of ovarian endometriomas, showing encouraging results in preserving ovarian reserve [14, 26]. Recently, Candiani et al. performed a randomized trial that compared cystectomy to ‘one-step’ CO_2_ fiber laser vaporization for the management of endometriomas with the aim to assess the impact of both techniques on ovarian reserve. The results suggested that ablation with CO_2_ laser technology was less harmful to the ovarian reserve of the operated ovary compared to cystectomy [27]. However, the same group did not later report a higher rate of natural conceptions or a favorable effect on IVF outcomes in CO_2_ laser treatment [28]. In our setting, even if we lack scientific evidence, we are confident that coagulation of the ovarian bed following stripping is presently used with more parsimony. The shift from stripping to ablation did not conversely occur. Only a small minority of women (3%) received ablation. This may be consequent to the inconclusive evidence available on the benefits of the ablation techniques. Of relevance here is that, even if some benefits of this approach on ovarian reserve were demonstrated, we lack evidence on the most relevant outcomes for women, i.e., improvement of pain, enhanced natural fertility and low rate of recurrence. To date, the stripping technique must yet be considered superior for these outcomes [19, 29]. Overall, our findings could be used to argue against the benefits of a more careful use of electric diathermy coagulation of the ovarian bed after or during stripping. Noteworthy, another interpretation could be claimed, i.e., that the observed damage following surgery may not be consequent to the excessive use of thermocoagulation.

In the debate regarding the improvement of the surgical technique, the mechanisms causing the damage are indeed still to be elucidated. Two main mechanisms have been postulated to cause the damage. The first claimed mechanism was the accidental removal of a consistent amount of ovarian tissue during cystectomy. Primordial follicles are found in more than 50% of the tissue removed [30]. Alternatively, the damage inflicted by surgery may be due to the local inflammation and/or vascular injury secondary to electrosurgical coagulation. Deckers et al. reported that bipolar electrocoagulation negatively impacts ovarian reserve compared to non-thermal hemostasis [31]. Overall, however, the clinical relevance of these supposed mechanisms remains undetermined [32]. In our opinion, this uncertainty has up to now hampered fruitful research on the technical tricks to improve the surgical technique.

The second main consequence of the raised awareness of post-surgical damage to ovarian reserve was the restriction in the indications to surgery. According to the latest ESHRE guidelines, surgery is now indicated only for larger cysts or in the absence of benefit of medical hormonal treatment on pain. Moreover, there is no indication to systematic surgery prior to ART [19]. This clinical view indirectly emerged also from our findings. Only a minority of infertile women were previously operated for endometriomas in our series (Fig. 1). Moreover, the dimension of the excised cyst was particularly interesting, the mean ± SD diameter being 47 ± 20 mm. The diameter exceeded 4 cm in 59% of women. In similar studies performed in our setting in 2003, 2005 and 2010, the mean ± SD diameter progressively increased, being 39 ± 15, 40 ± 24 and 42 ± 18 mm, respectively [2, 6, 33]. This observation suggests that a progressive restriction in the indications to surgery was already ongoing at that time and that it continued thereafter. Even if it is plausible that the magnitude of the damage may be related to the dimension of the excised cyst, there is yet no definite evidence on this issue. Our present study also failed to highlight a greater damage in those operated for larger cysts. Noteworthy, endometriomas larger than 4 cm could be detrimental even before surgery [34] and, therefore for larger cysts, it may become difficult to infer that post-surgical damage is exclusively related to surgical hits.

Some limitations of our study should be recognized. First, the study is retrospective, and therefore inevitably exposed to the inaccuracies of this study design. It has however to be pointed out that in previous studies performed on this topic in our setting, we did not observe main differences between retrospective and prospective studies [6, 33–35]. Second, our findings may reflect local rather than general situations. Forty percent of the women were operated in our hospital and the use of cyst wall ablation was very uncommon. Third, even if the sample size was remarkable, it did not allow reliable subgroup analyses for some of the variables, such as the technique used. Fourth, the exclusive inclusion of infertile women inevitably introduced a selection bias. Even if we are tempted to infer our findings to the whole group of women operated for endometriomas, this cannot be done. Noteworthy, while ovarian reserve is important for IVF success [36], it is poorly relevant for natural conception [37, 38]. Fifth, the wide mean time from surgery to IVF may be debatable. The possible explanation of this range is that he most of patients underwent surgery for the dimension of the cysts and not strictly for infertility. However, it is important to specify that in our hospital salpingocromoscopy is routinely performed during laparoscopy and patients may be induced to pursuit spontaneous pregnancy after surgery. Finally, failure to detect follicular growth during ovarian stimulation cannot be used to state that ovarian reserve is definitively compromised. This total lack of response may not be observed in a second cycle, and one cannot exclude that the use of a higher dosage of gonadotropins could allow to obtain some follicular growth. More in general, the exclusive use of ovarian response as a surrogate measurement of ovarian reserve is a possible concern. However, serum AMH could not be used because it does not allow to discern the relative contribution of the two ovaries and AFC is less reliable in operated ovaries [39].

In conclusion, the grown awareness on the detrimental impact of surgery on ovarian reserve has determined a restriction in the indications to surgery. However, operated women still face a relevant damage to their ovarian reserve. We plea for more research on the pathogenetic mechanisms causing the damage since this is a crucial preliminary necessary step. When these mechanisms will be definitely revealed, large randomized controlled trials could be designed to investigate possible benefits of changes in the surgical technique. Notably, future studies should assess not only ovarian reserve but also key clinical outcomes including pain relief, spontaneous pregnancy rates, and disease recurrence.

## Data Availability

No datasets were generated or analysed during the current study.
